# Calcium-Enriched Animal Manure Alleviates the Adverse Effects of Salt Stress on Growth, Physiology and Nutrients Homeostasis of *Zea mays L.*

**DOI:** 10.3390/plants8110480

**Published:** 2019-11-07

**Authors:** Bushra Niamat, Muhammad Naveed, Zulfiqar Ahmad, Muhammad Yaseen, Allah Ditta, Adnan Mustafa, Munazza Rafique, Riffat Bibi, Nan Sun, Minggang Xu

**Affiliations:** 1 Institute of Soil and Environmental Sciences, University of Agriculture, Faisalabad 38040, Pakistan; Bushraniamat77@gmail.com (B.N.); zulfiqar1409@gmail.com (Z.A.); dr.yaseen@gmail.com (M.Y.); adnanmustafa780@gmail.com (A.M.); 2 Department of Environmental Sciences, Shaheed Benazir Bhutto University Sheringal, Upper Dir, Khyber Pakhtunkhwa 18000, Pakistan; ad_abs@yahoo.com; 3 School of Biological Sciences, The University of Western Australia, 35 Stirling Highway, Perth, WA 6009, Australia; 4 National Engineering Laboratory for Improving Quality of Arable Land, Institute of Agricultural Resources and Regional Planning, Chinese Academy of Agricultural Sciences, Beijing 100081, China; sunnan@caas.cn; 5 Soil Bacteriology Section, Agri. Biotechnology Research Institute, AARI, Faisalabad 38000, Pakistan; 6 Soil and Water Conservation Research Institute (SAWCRI), Chakwal 48630, Pakistan; riffat_ises@yahoo.com

**Keywords:** compost, salt stress, sodicity, degradation, maize yield, Ca-fortified compost

## Abstract

Soil salinity and sodicity are among the main problems for optimum crop production in areas where rainfall is not enough for leaching of salts out of the rooting zone. Application of organic and Ca-based amendments have the potential to increase crop yield and productivity under saline–alkaline soil environments. Based on this hypothesis, the present study was conducted to evaluate the potential of compost, Ca-based fertilizer industry waste (Ca-FW), and Ca-fortified compost (Ca-FC) to increase growth and yield of maize under saline–sodic soil conditions. Saline–sodic soil conditions with electrical conductivity (EC) levels (1.6, 5, and 10 dS m^−1^) and sodium adsorption ratio (SAR) = 15, were developed by spiking soil with a solution containing NaCl, Na_2_SO_4_, MgSO_4_, and CaCl_2_. Results showed that soil salinity and sodicity significantly reduced plant growth, yield, physiological, and nutrient uptake parameters. However, the application of Ca-FC caused a remarkable increase in the studied parameters of maize at EC levels of 1.6, 5, and 10 dS m^−1^ as compared to the control. In addition, Ca-FC caused the maximum decrease in Na^+^/K^+^ ratio in shoot up to 85.1%, 71.79%, and 70.37% at EC levels of 1.6, 5, and 10 dS m^−1^, respectively as compared to the control treatment. Moreover, nutrient uptake (NPK) was also significantly increased with the application of Ca-FC under normal as well as saline–sodic soil conditions. It is thus inferred that the application of Ca-FC could be an effective amendment to enhance growth, yield, physiology, and nutrient uptake in maize under saline–sodic soil conditions constituting the novelty of this work.

## 1. Introduction

Soil salinization and sodication have been regarded as leading abiotic ecological constraints limiting the sustainable production of crops. These constraints have been extensively disseminated throughout the world, affecting about 20% of the total, and 33% of irrigated agricultural land [1]. In Pakistan, nearly 26% of the agricultural land in irrigated areas have been affected by salt stress; out of which about 56% are saline–sodic [2]. Under arid to semi-arid climatic conditions, salinization is caused by an increased accumulation of soluble salts in soil solution due to low rainfall and high temperatures [3].

Soil degradation through salinization and sodication is of prodigious apprehension severely affecting various domains of agriculture [4]. In arid or semi-arid climates, extreme evaporation results in the accumulation of salts in the upper soil layer, due to capillary action. This situation in surface soils severely affects soil structure and its hydraulic conductivity [3]. High amounts of exchangeable sodium and higher values of pH decrease soil water permeability which decreases water holding capacity of the soil and ultimately leads to a decrease in crop yields. Salt injuriousness and deprivation of essential plant nutrients are the two foremost causes for low production of crops under saline–sodic soil conditions [5]. Therefore, it is essential to determine economical, efficient, and sustainable management practices in order to lessen root zone salt damage caused by Na^+^ build up under saline–sodic soil conditions [6].

Various management practices have been tested to recover such types of salt-exaggerated soils; these include the development of salt resistant/tolerant varieties through breeding strategies [7] and the application of Ca-based chemical amendments especially gypsum, calcium chloride, etc. [8]. The former breeding approach is expensive and time-consuming, and even salt resistant/tolerant varieties require proper management practices for obtaining optimum crop yield under salt-exaggerated soil conditions. The latter approach directly supplies Ca^2+^ and seems applicable, depending upon the chemical being used (e.g., calcium chloride (CaCl_2_·2H_2_O) and gypsum (CaSO_4_·2H_2_O)). Ca-sources are known as soil modifiers as these restrict Na^+^ build up in the rhizosphere which severely affects plant growth and production under saline–sodic soils [9,10]. Earlier, Ghafoor et al. [11] found that application of Ca^2+^ sources are required for the recovery of saline–sodic soils in Pakistan. Recently an increased accumulation of Ca^2+^ ions in plant cells has been observed due to the application of Ca-based materials which help plants to cope with Na^+^-mediated stress under salt affected soil conditions [12,13]. Therefore, Ca-sources could be used to alleviate the deleterious impacts of soil salinity and sodicity for optimum production of various crop plants.

Nevertheless, while Ca-based amendments have the ability to recover the structure of soil, there have been some issues with the use of these amendments. For example, these amendments do not contribute towards microbial respiration or enzyme activities in soil; more salts are being added in soil solution with a temporary impact on soil properties under saline–sodic conditions. Moreover, with the application of Ca-based amendments, a large amount of good quality irrigation water is required which is deficient under arid to semi-arid soil conditions in order to solubilize the amendment itself and for the leaching of salts, which limits the practical application of such soil ameliorants.

In this regard, various organic approaches such as the use of mulch, manures (farmyard manure and green manure), and composts have been explored for their efficacy under saline–sodic soil conditions [14,15]. In spite of their efficacy in improving soil physicochemical (water holding capacity, cation exchange capacity, and plant nutrition elements) and biological properties (organic carbon, soil enzyme activities), these amendments have very little influence on the alleviation of soil salinity and sodicity stress when applied alone [16,17].

Therefore, it is imperative to use both Ca-based chemical and organic amendments in order to alleviate salt stress along with an improvement in soil quality and productivity under saline–sodic soil conditions which would ultimately lead to the improved growth and productivity of crops. Based on this hypothesis, the present study was conducted to find out the impact of compost, Ca-based fertilizer industry waste (Ca-FW), and Ca-fortified compost (Ca-FC) on the growth, and physiological and biochemical parameters of maize under saline–sodic soil conditions. To the best of our knowledge, none of the studies has reported the effect of Ca-fortified compost (Ca-FC) on growth and productivity of maize under saline–sodic soil conditions.

## 2. Results

### 2.1. Plant Growth Parameters

**Figure 1:**
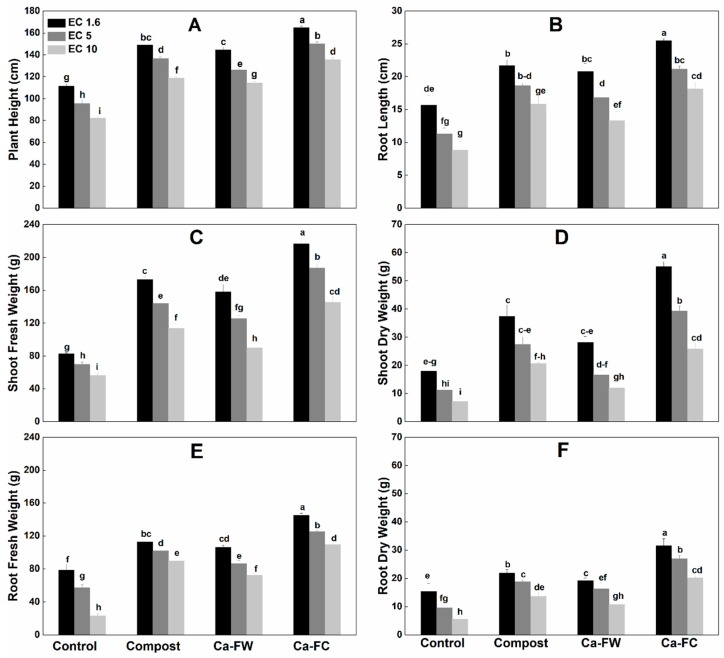
Effect of compost, Ca-based fertilizer industry waste (Ca-FW), and Ca-fortified compost (Ca-FC) on growth parameters of maize (*Zea mays* L.) under saline–sodic soil conditions. (Note: EC = electrical conductivity. Quantities sharing similar letters are statistically different from each other *at p* ≤ 0.05).

A statistically significant increase in plant growth parameters like plant height, shoot fresh and dry weight, root length, root fresh and dry weight, stem diameter, number of leaves per plant, and leaf area (Figure 1A–F, Table 1) was observed with the application of Ca-fortified compost (Ca-FC) under normal as well as under saline–sodic soil conditions as compared to the control and individual application of compost or Ca-based fertilizer industry waste (Ca-FW). The maximum shoot fresh weight was observed with the application of Ca-FC under normal as well as saline–sodic soil conditions as compared to the control and individual application of compost or Ca-FW (Figure 1). Further, individual application of compost yielded a better outcome compared to the control treatment, as an increase of 58.81%, 45.71%, and 36.68% in shoot fresh weight was recorded at electrical conductivity (EC) levels of 1.6, 5, and 10 dS m^−1^, respectively. Individual application of Ca-FW resulted in a 28.22%, 24%, and 23.33% increase in shoot fresh weight at EC levels of 1.6, 5, and 10 dS m^−1^, respectively. Similarly, application of Ca-FC caused the maximum increase in shoot dry weight up to 180.80%, 139.75%, and 104.13% at EC levels of 1.6, 5, and 10 dS m^−1^, respectively as compared to the control treatment. It was followed by the individual application of compost which increased shoot dry weight by 95.32%, 88.95%, and 41.98% and Ca-FW by 70.50%, 66.16%, and 24.22% at EC levels of 1.6, 5, and 10 dS m^−1^, respectively as compared to the control treatment.

Regarding plant height, the application of Ca-FC caused a significant increase in plant height (i.e., 63.33%, 58.5%, and 53.66% at EC levels of 1.6, 5, and 10 dS m^−1^, respectively) as compared to the control treatment. It was followed by the individual application of compost and Ca-FW. An increase of 51%, 39%, and 25.33% in plant height compared to the control treatment, was recorded with the individual application of compost at EC levels of 1.6, 5, and 10 dS m^−1^, respectively. Similarly, application of Ca-FW alone increased plant height up to 50%, 45.83%, and 35.66% at EC levels of 1.6, 5, and 10 dS m^−1^, respectively as compared to the control treatment.

In the same way, the individual application of compost increased root length up to 79.31%, 64.75%, and 38.27% at EC levels of 1.6, 5, and 10 dS m^−1^, respectively in comparison with the control treatment. Root length increased up to 51%, 48.57%, and 32.95% at EC levels of 1.6, 5, and 10 dS m^−1^, respectively as compared to the control treatment when Ca-FW was applied separately. However, the application of Ca-FC caused the maximum increase in root length by 105.74%, 86.82%, and 62.73% at EC levels of 1.6, 5, and 10 dS m^−1^, respectively as compared to the control treatment. Similarly, application of Ca-FC maximally enhanced the root fresh weight up to 175.30%, 98.67%, and 77.89% at EC levels of 1.6, 5, and 10 dS m^−1^, respectively in comparison with the control treatment. Root fresh weight was increased up to 120.14%, 66.2%, and 51.21% at EC levels of 1.6, 5, and 10 dS m^−1^, respectively as compared to the control when compost was applied alone. A similar trend was observed in the case of root dry weight.

In the case of stem diameter, application of compost or Ca-FW alone showed a small increase at EC levels of 1.6, 5, and 10 dS m^−1^ compared to the control (Table 1). The maximum increase in stem diameter was observed with the application of Ca-FC (i.e., 68.21%, 64.10%, and 51.70% at EC levels of 1.6, 5, and 10 dS m^−1^, respectively) as compared to the control treatment. Likewise, application of Ca-FC showed the maximum increase in the number of plant leaves by 57.97%, 52.42%, and 45.87% over the control treatment at EC levels of 1.6, 5, and 10 dS m^−1^, respectively. Moreover, application of Ca-FC caused the maximum and statistically significant improvement in leaf area by 51.21%, 49.23%, and 47.86% at EC levels of 1.6, 5, and 10 dS m^−1^, respectively as compared to the control treatment.

### 2.2. Yield Parameters and Protein Contents

Like the growth parameters, yield parameters such as cob size, cob fresh weight, cob dry weight, and 1000-grains weight was significantly reduced under saline-sodic soil conditions without any treatment (Table 1 and Table 2). The maximum and statistically significant increase was noted with the application of Ca-FC under normal as well as saline–sodic soil conditions. The application of Ca-FC increased cob size by 134.68%, 118.55%, and 114.73% at EC levels of 1.6, 5, and 10 dS m^−1^, respectively as compared to the control treatment. A minor increase in cob size was observed by the individual application of compost and Ca-FW at EC levels of 1.6, 5, and 10 dS m^−1^. In the case of cob fresh weight, individual application of compost increased 69.37%, 51.42%, and 46.66% at EC levels of 1.6, 5, and 10 dS m^−1^, respectively as compared to the control treatment. However, the maximum cob fresh weight was observed by the application of Ca-FC with an increase of 99.99%, 81.46%, and 70% in cob fresh weight at EC levels of 1.6, 5, and 10 dS m^−1^, respectively compared to the control treatment. Likewise, application of Ca-FC markedly increased cob dry weight by 72.30%, 67.84%, and 66.66% at EC levels of 1.6, 5, and 10 dS m^−1^, respectively as compared to the control treatment. Furthermore, application of compost alone slightly increased 1000-grains weight by 16.25%, 15.30%, and 13.76% at EC levels of 1.6, 5, and 10 dS m^−1^, respectively than the control treatment, while the application of Ca-FW showed the maximum increase in 1000-grains weight by 29.01%, 22.58%, and 20.63% at EC levels of 1.6, 5, and 10 dS m^−1^, respectively. Data regarding protein contents indicated that application of Ca-FC caused the maximum increase up to 173.33%, 162.22%, and 155.15% at EC levels of 1.6, 5, and 10 dS m^−1^, respectively more than the control treatment (Table 2).

### 2.3. Physiological Parameters 

**Figure 2:**
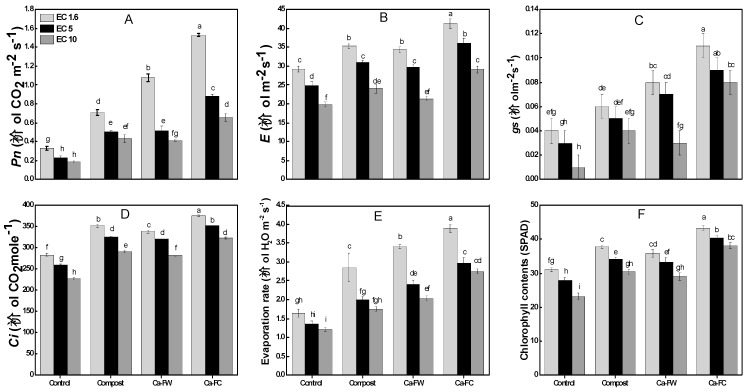
Effect of compost, Ca-FW, and Ca-FC on physiological parameters of maize (*Zea mays* L.) under saline–sodic soil conditions. (Note: Ca-FW = calcium based fertilizer industry waste; Ca-FC = calcium-fortified compost; EC = electrical conductivity. Quantities sharing similar letters are statistically different from each other at *p* ≤ 0.05. *Pn* = photosynthetic rate; *E* = transpiration rate; *gs* = stomatal conductance; *Ci* = internal CO_2_ concentration; SPAD = Soil Plant Analysis Development chlorophyll meter).

Data regarding physiological parameters (Figure 2A–F) indicate that individual application of compost caused a slight increase in photosynthetic rate by 19%, 11%, and 6% at EC levels of 1.6, 5, and 10 dS m^−1^, respectively, compared to the control treatment. However, the application of Ca-FC caused the maximum increase in photosynthetic rate by 67%, 43%, and 35% at EC levels of 1.6, 5, and 10 dS m^−1^, respectively as compared to the control treatment. Similarly, the maximum increase in stomatal conductance was recorded with the application of Ca-FC (i.e., 56.16%, 42.39%, and 34.11% at EC levels of 1.6, 5, and 10 dS m^−1^, respectively in comparison to the control treatment. In the same way, application of Ca-FC caused the maximum increase in evaporation rate up to 138.03%, 129.16%, and 116.78% at EC levels of 1.6, 5, and 10 dS m^−1^, respectively as compared to the control treatment. A similar trend was observed in the case of transpiration rate. Likewise, the maximum increase in internal CO_2_ concentration was observed with the application of Ca-FC by 42.145, 35.68%, and 32.62% at EC levels of 1.6, 5, and 10 dS m^−1^, respectively compared to the control treatment.

The maximum chlorophyll contents were recorded with the application of Ca-FC by a 64.22%, 45.32%, and 39.77% increase over the control at EC levels of 1.6, 5, and 10 dS m^−1^, respectively. 

#### Water Relations

**Figure 3:**
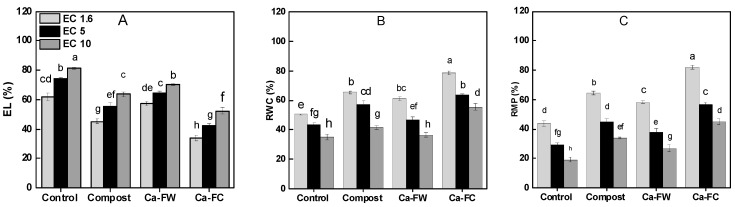
Effect of compost, Ca-FW, and Ca-FC on physiological parameters of maize (*Zea mays* L.) under saline–sodic soil conditions. (Note: Ca-FW = calcium based fertilizer industry waste; Ca-FC = calcium-fortified compost; EL = electrolyte leakage; RWC = relative water contents; RMP = relative membrane permeability. Quantities sharing similar letters are statistically different from each other at *p* ≤ 0.05).

Considerable reductions in maize water relations in terms of relative water contents, relative membrane permeability, electrolyte leakage, and osmotic potential were observed under saline–sodic soil conditions. In the case of relative water contents (RWC), application of Ca-FW alone caused the minimum increase at EC levels of 1.6, 5, and 10 dS m^−1^, respectively. Nonetheless, application of Ca-FC caused the maximum increase in RWC by 18.21%, 13.27%, and 12.12% at EC levels of 1.6, 5, and 10 dS m^−1^, respectively as compared to the control treatment (Figure 3B). Likewise, the maximum increase in relative membrane permeability was observed with the application of Ca-FC up to 132.54%, 93.75%, and 87.37% compared to the control at EC levels of 1.6, 5, and 10 dS m^−1^, respectively (Figure 3C). A similar trend was observed with the application of Ca-FC in the case of osmotic potential under normal as well as saline–sodic soil conditions (Table 2). Furthermore, application of Ca-FC considerably decreased electrolyte leakage by 45%, 43%, and 36% at EC levels of 1.6, 5, and 10 dS m^−1^, respectively in comparison to the control treatment (Figure 3A). 

### 2.4. Chemical Parameters

**Figure 4:**
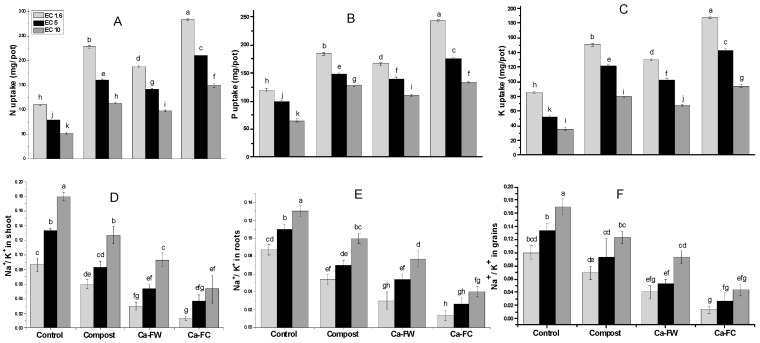
Effect of compost, Ca-FW, and Ca-FC on nutrients homeostasis of maize (*Zea mays* L.) under saline–sodic soil conditions. (Note: Ca-FW = calcium based fertilizer industry waste; Ca-FC = calcium-fortified compost. Quantities sharing similar letters are statistically different from each other at *p* ≤ 0.05.

A statistically significant effect regarding N, P, and K uptake was recorded with the application of Ca-FC under normal as well as saline–sodic soil conditions (Figure 4A–C). The maximum N uptake in the shoot was observed with the application of Ca-FC (186.01%, 168.10%, and 157.74% at EC levels of 1.6, 5, and 10 dS m^−1^, respectively) in comparison to the control treatment. A similar trend was observed in the case of P uptake. Application of Ca-FC caused a significant increase in P uptake by 106.67%, 102.50%, and 78.71% at EC levels of 1.6, 5, and 10 dS m^−1^, respectively as compared to the control. In the same way, application of Ca-FC markedly improved K uptake up to 173.90%, 162.96%, and 119.93% at EC levels of 1.6, 5, and 10 dS m^−1^, respectively compared to the control treatment.

Conversely, it was found that the Na^+^/K^+^ ratio in root significantly decreased by 85.19%, 75.76%, and 69.23% by Ca-FC treatment over the control at EC levels of 1.6, 5, and 10 dS m^−1^, respectively (Figure 4E). Similarly, the maximum decrease (i.e., 85.1%, 71.79%, and 70.37%) in the Na^+^/K^+^ ratio of shoot was observed with the application of Ca-FC at EC levels of 1.6, 5, and 10 dS m^−1^, respectively as compared to control treatment (Figure 4D). Similarly, application of Ca-FC caused a maximum decrease in Na^+^/K^+^ ratio (i.e., 74.36%, 60.26%, and 50.55%) in grains compared to the control at EC levels of 1.6, 5, and 10 dS m^−1^, respectively (Figure 4F).

## 3. Discussion

Soil salinization is a notorious, rather classical, but sometimes emergent, issue for agricultural production. In arid and semi-arid regions of the world, most of the soils are salt-affected with low agricultural potential [18]. In order to exploit salt-exaggerated soils, various strategies such as the use of good quality water, good drainage, suitable cultural practices, and the addition of appropriate soil ameliorants have been found suitable [6,11]. Various researchers have found that use of soil amendments such as gypsum containing Ca^2+^ can compete with Na^+^ on the exchangeable sites and ultimately ameliorate the impact of salinity and sodicity. In this regard, the present study was conducted using Ca sourced from a Ca-based fertilizer industry waste (Ca-FW) and mixing it with organic wastes and composting both for seven days.

Salt stress interrupts the growth of plants via osmotic stress, specific ion toxicity, and nutritional imbalance [1]. In the present study, all the examined growth parameters were significantly reduced under saline–sodic soil conditions (Figure 1, Table 1, Table 2 and Table 3). Through the application of organic (compost) and inorganic (i.e., Ca-based fertilizer industry waste (Ca-FW)) amendments, a positive effect on growth parameters under normal as well as saline–sodic soil conditions were observed. More specifically, the observed growth and yield parameters like plant height, shoot dry weight, root dry weight, cob dry weight, and 1000-grains weight were significantly improved by the application of Ca-fortified compost (Ca-FC) at all EC levels (i.e., 1.6, 5, and 10 dS m^−1^) in comparison to the control treatment. Earlier, it was determined that water holding capacity, cation exchange capacity, macronutrients as well as micronutrients availability, and soil physical attributes were improved with the addition of organic amendments such as compost under normal as well as saline–sodic soil conditions [19,20,21]. Improvement in soil physicochemical properties through organic material addition might enhance the physiological functions of the plant and ultimately the growth and yield parameters noted during this study [17,22,23]. It may also be due to the role of organic amendments in improving soil biological activities which are directly involved in nutrient uptake and ultimately improved growth and yield parameters [15,22]. Earlier, it was determined that the application of organic amendments under normal as well as under salt stress conditions, significantly improved soil microbial activity. The improvement in soil microbial activity enhances soil nutrients mineralization and ultimately improves nutrient availability and their uptake in crop plants [24,25]. 

In the current study, physiological parameters like chlorophyll contents, relative water contents, relative membrane permeability, photosynthetic rate, transpiration rate, evaporation rate, etc. were increased with the application of Ca-FC under normal and saline–sodic soils. Under salinity stress, chlorophyll contents as well as the photosynthetic capacity of crop plants decreased [26,27]. It also was revealed that a decline in mesophyll conductance to carbon dioxide (CO_2_) occurs under salt stress [28]. In the present study, application of compost, Ca-FW, and Ca-FC significantly improved the chlorophyll contents under salt stress which might be due to the increased availability of nutrients with the application of compost (Figure 2).

It is well documented that the uptake of essential nutrients like potassium, calcium, and nitrogen decreases due to increased sodium (Na^+^) concentration in soil solution [5] under saline conditions. However, compost application significantly decreased Na^+^ and increased K^+^ uptake in the present study. Nevertheless, in the current study, maximum sodium concentration in root, shoot, and grains was observed in the control treatment and minimum sodium concentration in root, shoot, and grains was observed with the application of Ca-FC at all EC levels. Earlier, compost and Ca-products have been found to be more effective in mitigating the adverse effects of Na^+^ in plants [11]. Moreover, the application of composted municipal solid waste (MSW) helped in the release of Ca^2+^ from the applied Ca-based amendments such as gypsum [17]. Therefore, application of Ca-FW and Ca-FC as tested in the present study might have served as available Ca sources. The Ca released during the composting of organic amendments combined with Ca-FW would ultimately result in the flocculation of Na^+^ present.

In the present study, the maximum K^+^ uptake in maize shoot was recorded with the application of Ca-FC under normal as well as saline–sodic soil conditions. Earlier, it was observed that K^+^ uptake enhanced in shoot under salinity stress by applying organic amendments like compost [29] which might be due to the stimulatory effect of organic materials on the cation exchange capacity (CEC) of soil. In a precise manner, application of organic amendments (e.g., compost or poultry manure) increases the CEC of the soil, thereby limiting the entry of Na^+^ to the exchangeable site and ultimately leading to a better uptake of both soluble and exchangeable K^+^ [15,16,22]. Moreover, application of compost increases nutrients availability especially K^+^, Mg^2+^, and Ca^2+^ via maintaining a better nutrient equilibrium with the soil solution.

The increase in N, P, and K uptake noted in the present study might be due to the indirect improvement of soil physicochemical properties. Earlier, it was found that long term application of Ca-amendments plays an important role in improving soil organic carbon (SOC) stocks [30,31]. Moreover, mineral availability in the form of Ca-amendments has been found to be a key regulator of soil carbon [32,33,34]. Improvement in SOC contents has a direct effect on soil physicochemical properties and ultimately an increased supply of macro- and micronutrients to crop plants [21]. Other researchers have also noted an increase in soil organic carbon contents with the application of Ca-amendments [31,35]. In case of the application of organic amendments, these have been found to increase the aggregate stability of the soil which ultimately results in higher storage of soil organic carbon, a positive effect on soil physiochemical properties, and ultimately better nutrient availability and uptake [31,36].

In the present study, Na^+^/K^+^ ratio in the root, shoot, and grains was significantly decreased with the application of Ca-FC under normal as well as saline–sodic soil conditions which might be due to the limited entry of Na^+^ into the plant (Figure 2). Earlier, it was suggested that application of organic amendments (farmyard manure and green manure) might be used as chelating agents for decontamination of lethal salts especially Na^+^ and Cl^−^ ions [17,22]. The organic amendments and gypsum were found to increase the growth and productivity of various crops (e.g., rice, wheat, sugarcane, cotton, and tomatoes [9,11,17,22]). As earlier mentioned, the improved availability of Ca^2+^ and other nutrients with the application of Ca-FC might have helped with the improved growth and productivity of maize in the present study. Recently, the combined application of green waste compost, sedge peat, and furfural residue (1:1:1 on volume basis) significantly reduced Na^+^ content and improved CEC and availability of nutrient uptake (NPK) contents [6,15].

Hu and Schmidhalter [37] observed reduced availability of macronutrients especially NPK under saline stress conditions. In the current study, maximum phosphorus uptake in maize shoot was observed with the application of Ca-FC (Figure 1). Application of compost has been reported to lower pH which enhances the availability of essential plant nutrients under saline stress conditions [38]. Moreover, the application of compost has been directly involved in the provision of plant nutrients [20,21]. Various researchers around the world have found that the application of organic amendments significantly improve the physicochemical properties of the soil which ultimately improves the availability of various nutrients [9,17,21]. 

The present study describes the response of compost, Ca-FW, and Ca-FC to maize crop growth, development, and nutrients homeostasis under saline–sodic soil conditions. Overall this study implies that the application of Ca-FC significantly enhanced growth, physiology, grain quality, and NPK uptake of maize by restoring the negative impacts caused by salt-stress. 

## 4. Materials and Methods

### 4.1. Preparation of Calcium-Fortified Compost

Animal manure was collected from the Directorate of Farms, University of Agriculture Faisalabad, Pakistan for compost preparation. Ca-based fertilizer industry waste (Ca-FW) was collected from the calcium ammonium nitrate fertilizer production industry (Pak Arab Fatima Fertilizers, Ltd. Multan, Pakistan) and it contained about 10% Ca and 24% nitrogen. Calcium-fortified compost (Ca-FC) was prepared by incubating calcium (Ca)-based fertilizer industry waste (Ca-FW) in the animal manure at the rate of 50:50 (w/w) with 0.1% molasses. The material was thoroughly mixed and composted by continuously running a locally fabricated composter (500 kg capacity) for seven days [19,20,21]. Simple compost with 0.1% molasses was also prepared in a similar way without Ca-FW. The chemical analysis results of normal compost and Ca-FC regarding various nutrient parameters were done through the standard procedure given by Nelson and Sommers [39] and Ryan et al. [40] and the results are presented in Table 3. Calcium contents in the digested sample of compost and Ca-FC were determined through an atomic absorption spectrophotometer (Hitachi Zeeman, Japan) equipped with a graphite furnace with a Ca-cathode lamp.

### 4.2. Pot Experiment

A pot experiment was conducted in the greenhouse of the Institute of Soil and Environmental Sciences (ISES) at the University of Agriculture, Faisalabad (UAF), Pakistan using a maize variety (Malka 2015) kindly provided by Maize Research Section, Ayub Agricultural Research Institute Faisalabad, Pakistan as a test crop. The soil was taken from the research area of ISES, UAF, air dried, ground, and sieved (2 mm). Each pot (25 cm in diameter and 23 cm high) was filled with 8 kg of soil. The soil was analyzed for various physicochemical properties following the methods described by Ryan et al. [40]. The soil analysis showed that the textural class of the soil was sandy clay loam (sand = 50%, silt = 35%, clay = 15%) with a saturation percentage of 32% and ECe = 1.637 dS m^−1^. The soil was slightly alkaline in nature having pH = 7.33, soluble CO_3_^2-^ = 0.88 mmol_c_ L^−1^ and soluble HCO_3_^2-^ = 0 mmol_c_ L^−1^, CEC = 11.8 cmol_c_ kg^−1^, and organic matter = 0.72%.

In the present study, there were two factors: electrical conductivity with three levels (1.6, 5, and 10 dS m^−1^) and treatment at four levels (control, compost, Ca-FW, and Ca-FC). The saline–sodic soil was made by adding a mixture of salts in soil. There were three levels of electrical conductivity (EC)—1.6 dS m^−1^ (original/ normal soil EC), 5 and 10 dS m^−1^ (saline–sodic)—and sodium adsorption ratio (SAR) was fixed at 15 under both EC levels. For each replication of twelve treatments, the saline–sodic soil was developed separately. To prepare saline–sodic soil, about 0.66, 2.16, 0.72, and 0.23 g of NaCl, Na_2_SO_4_, CaCl_2_, and MgSO_4_, respectively were homogeneously mixed in 32 kg of soil to develop an EC level of 5 dS m^−1^ and SAR of 15. In order to develop an EC level 10 dS m^−1^ and SAR of 15, about 1.18, 5.94, 3.6, and 1.3 g of NaCl, Na_2_SO_4_, CaCl_2_, and MgSO_4_, respectively were homogeneously mixed in 32 kg of soil. Compost, Ca-FW, and Ca-FC were applied at the rate of 1% w/w. Overall, there were 36 pots arranged in a completely randomized design (CRD) with three replications. In each treatment, the recommended rate of chemical fertilizer (NPK = 120:80:60 kg ha^−1^) was applied. Three seeds per pot were sown, and after germination one plant per pot was maintained through thinning. Recommended agronomic practices like thinning, hoeing, etc. were followed. 

### 4.3. Data Collection

#### 4.3.1. Physiological Parameters

Infrared gas analyzer (IRGA) was used to measure photosynthetic rate, stomatal conductance, transpiration rate, evaporation rate, and internal CO_2_ concentrations. Two leaves of a single plant from each pot were selected to record the data regarding the above-mentioned parameters. Chlorophyll contents were measured as SPAD (Soil Plant Analysis Development) value from the third upper leaf using chlorophyll meter SPAD-502 [41]. Relative water content (RWC), relative membrane permeability (RMP), and electrolyte leakage (EL) were measured by using entirely stretched flag leaves.

In order to measure relative water content (RWC), leaves were placed in a refrigerator for 24 h at 4 °C after cutting and sealing in a plastic bag and fresh weight was measured. Fully turgid weight was measured after soaking leaves in distilled water, while dry weight was measured after drying leaves in an oven at 72 °C for 24 h. The following equation was used to calculate RWC as defined by Teulat et al. [42].

(1) $$RWC\;\left(\%\right)=\frac{Fresh\;wieght-Dry\;weight}{Turgid\;weight-Dry\;weight}\times 100$$

For computing electrolyte leakage (EL), even leaf discs were placed in test tubes and 50 mL distilled water was added to each tube. Later, each test tube was shaken for 4 h at room temperature and electrical conductivity (EC_1_) of the solution was measured by an EC meter. Then, the test tubes with samples were autoclaved and after cooling, a second reading of electrical conductivity (EC_2_) was measured. Electrolyte leakage was calculated using the following formula [43]:

(2) $$EL\;\left(\%\right)=\;\frac{EC_{1}}{EC_{2}}\;\times 100$$

To quantify relative membrane permeability (RMP), leaves were cut and shifted into test tubes with 20 mL distilled water. The test tubes were vortexed for 10 s and EC_0_ was measured using an EC meter. Another EC meter reading (EC_1_) was taken after the test tubes were subsequently vortexed for 24 h at 4 °C. The samples in the test tubes were autoclaved at 121 °C for 20 min and EC meter reading (EC_2_) was measured. The following formula was used to calculate RMP [44]:

(3) $$RMP\;\left(\%\right)=\;\frac{EC_{1}-EC_{0}}{EC_{2}-EC_{0}}\;\times 100$$

In order to quantify osmotic potential, flag leaves were placed in the freezer for 1­­–2 days. After freezing, leaves were folded, kept in a 1.5 mL Eppendorf tube, and pinched with a needle so that leaf sap could be taken. Leaf sap was taken with a pipette and stored in another Eppendorf tube (0.5 mL). After that, readings of these samples regarding osmotic potential were recorded with the help of a cryoscopic osmometer [45].

#### 4.3.2. Plant Growth and Yield Parameters

The plants were harvested 120 days after sowing and the data of plant height and fresh weight of root and shoot were measured using a meter rod and digital weighing balance. Cobs were detached manually, and their fresh weight was measured. The size of each cob as cob length was measured with the help of a meter rod after harvesting. Then, these samples were washed with deionized water and exposed to air under the shade. Samples were placed in forced air driven oven (Tokyo Rikakikai, Eyela WFO-600 ND, Tokyo, Japan) at 80 °C for 48 h until constant weight was achieved to quantify root dry weight (RDW), shoot dry weight (SDW), and cob dry weight. After drying, grains were detached from cobs and 1000-grains weight was measured using the digital weighing balance. Length and width of leaves were recorded with the help of measuring tape and then leaf area (cm^2^) was measured by multiplying both length and width. Stem girth/diameter (cm) of plants from each pot was measured with the help of Vernier caliper at time of harvesting.

#### 4.3.3. Chemical Parameters

Different chemical parameters of plants such as nitrogen, phosphorus, potassium, and sodium were analyzed after wet digestion. Shoot, root, and grain samples were air-dried and then oven-dried at 65 °C and ground by a mechanical grinder to prepare the samples for chemical analysis. The Wolf [46] method was used for digestion in which the plant samples (0.5 g) were placed in digestion tubes and allowed to stand overnight after the addition of sulfuric acid (10 mL). On the next day, hydrogen peroxide (2 mL) was added and the digestion tubes were placed on a hot plate maintaining a temperature up to 300–350 °C. Hydrogen peroxide (2 mL) was added again and again until the black color disappeared, and a colorless solution was obtained. The samples in the digestion tubes were cooled down at room temperature and distilled water was added until the total volume was 50 mL. The sample dilutions were filtered through Whatman 42 paper and the filtrate was stored in plastic bottles before they were analyzed. Potassium and sodium were determined using a flame photometer, nitrogen by determined with the Kjeldahl apparatus, and phosphorus was measured by a spectrophotometer [47]. Then the uptake of nitrogen, phosphorus, and potassium was calculated using the following formula:

(4) $$\mathrm{NPK}\;\mathrm{uptake}\;\mathrm{in}\;\mathrm{shoots}\;(\mathrm{mg}\;pot^{-1})\;=\;\frac{\mathrm{NPK}\;\mathrm{concentration}\;\mathrm{in}\;\mathrm{shoots}\;(\%)\;\times \;\mathrm{Dry}\;\mathrm{matter}\;(\mathrm{mg}\;pot^{-1})}{100}$$

Then, the Na^+^/K^+^ ratio in root, shoot, and grains was also calculated.

#### 4.3.4. Biochemical Analysis

Crude protein contents in grains were determined by the multiplication of nitrogen contents obtained through Kjeldahl’s method in grains with a 6.25 factor [48].

#### 4.3.5. Statistical Analysis

Using the analysis of variance (ANOVA) technique, the collected data were analyzed at *p* ≤ 0.05 under a completely randomized design (CRD) with the help of Statistics 8.1 software and means were compared by using Tukey’s Honest Significant Difference (HSD) test [49]. All the graphs in the manuscript were drawn on Origin Pro version 9.1 computer based-software. 

## 5. Conclusions

It is summarized that application of Ca-FC significantly enhanced the growth, yield, physiology, and nutrient uptake in maize under normal and saline–sodic soil conditions as compared to the control and individual application of compost or Ca-based fertilizer industry waste (Ca-FW). Similarly, Ca-fortified compost (Ca-FC) significantly enhanced NPK uptake and reduced Na^+^/K^+^ ratio in the root, shoot, and grains of maize under normal as well as saline–sodic soil conditions as compared to the control and individual application of compost or Ca-FW. The approach is promising in ameliorating saline–sodic soils and enhancing maize productivity; however, multi-site field trials need to be performed to warrant successful performance in the field.

## Figures and Tables

**Table 1:** Effect of compost, Ca-FW, and Ca-FC on growth and yield parameters of maize (*Zea mays* L.) under saline–sodic soil conditions.

Treatments	Stem Diameter (cm)			No. of Leaves (plant^−1^)			Leaf Area (cm^2^)		
	**EC 1.6 dS m^−1^**	**EC 5 dS m^−1^**	**EC 10 dS m^−1^**	**EC 1.6 dS m^−1^**	**EC 5 dS m^−1^**	**EC 10 dS m^−1^**	**EC 1.6 dS m^−1^**	**EC 5 dS m^−1^**	**EC 10 dS m^−1^**
Control	9.67 ± 0.33efg	8.33 ± 0.33gh	7.33 ± 0.33h	8.00 ± 0.58de	7.00 ± 0.58ef	6.33 ± 0.33f	343.59 ± 1.32f	302.14 ± 1.05h	267.72 ± 1.59i
Compost	12.00 ± 0.58c	11.00 ± 0.00cde	10.00 ± 0.58def	10.67 ± 0.33ab	9.33 ± 0.33bcd	8.00 ± 1.15de	426.13 ± 3.69c	396.52 ± 4.63d	347.80 ± 2.11f
Ca-FW	11.33 ± 0.33cd	10.33 ± 0.33def	9.00 ± 0.58fg	10.33 ± 0.33ab	9.67 ± 0.33bc	8.67 ± 0.88cd	420.60 ± 3.82c	386.98 ± 4.03e	328.33 ± 2.98g
Ca-FC	14.67 ± 0.33a	13.67 ± 0.88ab	12.33 ± 0.33bc	11.67 ± 0.33a	10.67 ± 0.33ab	10.00 ± 0.58bc	508.04 ± 1.30a	450.91 ± 3.28b	404.83 ± 3.74d
	**Cob Size (cm)**			**Cob Fresh Weight (g)**			**Cob Dry Weight (g)**		
Control	11.33 ± 0.33fg	9.00 ± 0.58hi	7.67 ± 0.33i	150.00 ± 1.15e	127.67 ± 1.45f	106.67 ± 3.33g	85.00 ± 2.89d	66.00 ± 2.31e	49.33 ± 1.45f
Compost	16.67 ± 0.88cd	13.00 ± 0.58ef	11.33 ± 0.88fg	220.00 ± 5.77bc	193.33 ± 2.40d	180.67 ± 2.96d	115.00 ± 2.89b	76.00 ± 4.16d	66.00 ± 3.06e
Ca-FW	15.67 ± 0.33d	13.33 ± 0.88e	10.67 ± 0.67gh	208.33 ± 8.33c	180.67 ± 2.33d	156.33 ± 4.48e	103.33 ± 3.33c	65.33 ± 2.91e	53.00 ± 1.53f
Ca-FC	24.33 ± 0.33a	19.67 ± 0.88b	18.00 ± 0.58bc	255.00 ± 2.89a	231.67 ± 6.01b	213.33 ± 8.82c	142.67 ± 3.93a	110.00 ± 5.77bc	85.00 ± 2.89d

Note: Ca-FW = calcium based fertilizer industry waste; Ca-FC = calcium-fortified compost; EC = electrical conductivity. Quantities sharing similar letters are statistically different from each other at *p* ≤ 0.05.

**Table 2:** Effect of compost, Ca-FW, and Ca-FC on 1000-grains weight, osmotic potential, and protein contents of maize (*Zea mays* L.) under saline–sodic soil conditions.

Treatments	1000-Grains Weight (g)			Osmotic Potential (osmol/kg)			Protein (%)		
	**EC 1.6 dS m^−1^**	**EC 5 dS m^−1^**	**EC 10 dS m^−1^**	**EC 1.6 dS m^−1^**	**EC 5 dS m^−1^**	**EC 10 dS m^−1^**	**EC 1.6 dS m^−1^**	**EC 5 dS m^−1^**	**EC 10 dS m^−1^**
Control	258.82 ± 4.35f	249.46 ± 0.53g	229.62 ± 0.47i	0.37 ± 0.01de	0.33 ± 0.02fg	0.23 ± 0.02i	9.56 ± 0.10h	6.98 ± 0.46i	4.66 ± 0.70j
Compost	300.88 ± 0.73b	283.80 ± 1.08d	264.77 ± 1.13e	0.45 ± 0.01b	0.40 ± 0.01cd	0.31 ± 0.01gh	18.10 ± 0.05cd	16.25 ± 0.19e	14.10 ± 0.15f
Ca-FW	290.63 ± 1.33c	266.23 ± 0.87e	243.67 ± 1.32h	0.44 ± 0.01bc	0.38 ± 0.01de	0.29 ± 0.01h	16.69 ± 0.63de	14.20 ± 0.24f	12.00 ± 0.91g
Ca-FC	333.91 ± 1.87a	300.94 ± 0.81b	281.47 ± 0.94d	0.51 ± 0.01a	0.44 ± 0.01b	0.36 ± 0.01ef	25.31 ± 0.66a	22.60 ± 0.68b	19.19 ± 0.16c

Note: Ca-FW = calcium based fertilizer industry waste; Ca-FC = calcium-fortified compost; EC = electrical conductivity. Quantities sharing similar letters are statistically different from each other at *p* ≤ 0.05.

**Table 3:** The chemical analysis of normal compost and Ca-FC.

Parameters	Composted Organic Fertilizer	Ca-Based Fertilizer Industry Waste (Ca-FW)	Ca-Fortified Compost (Ca-FC) (50:50 w/w)
Carbon (g kg^−1^)	203 ± 16.6 ^†^	-	104.5 ± 9.2
Nitrogen (g kg^−1^)	15.8 ± 1.2	24 (%)	108 ± 4.3
Total P (g kg^−1^)	2.89 ± 0.9	-	1.76.0 ± 0.2
Olson P (mg kg^−1^)	247 ± 12	-	138 ± 8.7
Ca (g kg^−1^)	0.046 ± 0.002	10 (%)	48.3 ± 3.4
C:N	16.24 ± 1.2	-	14.05 ± 0.6
C:P	70.24 ± 5.9	-	9.19 ± 0.18
pH	6.43 ± 0.24	8.02	6.79 ± 0.13

Note = ^†^ shows the standard error of means (SEM) where *n* = 3.
